# Reproductive experiences and factors influencing contraceptive use among female head‐porters in Ghana: A cross‐sectional study

**DOI:** 10.1002/hsr2.1298

**Published:** 2023-06-01

**Authors:** Seth Amponsah‐Tabi, Kwadwo Sarbeng, Edward Dassah, Amponsah Peprah, Gerald Owusu Asubonteng, Frank Ankobea, Stephen Opoku, Ebenezer Senu, Emmanuel S. K. Morhe, Kwabena Antwi Danso

**Affiliations:** ^1^ Directorate of Obstetrics and Gynaecology Komfo Anokye Teaching Hospital Kumasi Ghana; ^2^ College of Health Sciences Kwame Nkrumah University of Science and Technology Kumasi Ghana; ^3^ Department of Molecular Medicine, School of Medicine and Dentistry Kwame Nkrumah University of Science and Technology Kumasi Ghana; ^4^ Kumasi Centre for Collaborative Research in Tropical Medicine Kumasi Ghana; ^5^ University of Health and Allied Sciences Ho Ghana

**Keywords:** contraceptive use, female head‐porters (Kayayei), reproductive experience, sexual partners

## Abstract

**Background and Aims:**

Female head‐porters are a cohort of women who have migrated from their rural communities into commercial cities in search of better economic opportunities. These young women are vulnerable to untoward reproductive experiences. The study assesses the reproductive experiences of women and the factors influencing contraceptive use among them.

**Methods:**

A cross‐sectional study was conducted from January to May 2021 in the Kumasi Metropolis (*n* = 280). The study included 280 female head‐porters within the reproductive age of 15–49 years. Convenience sampling and consecutive recruitment were used to obtain the needed sample size. All statistical significance was declared at a *p*‐value of <0.05.

**Results:**

Forty‐two percent of respondents had a history of contraceptive use (all modern or artificial contraception). The study found gravidity (*p* < 0.0001), parity (*p* < 0.0001), number of sexual partners post‐migration (*p* = 0.008), and age of first sex (*p* = 0.033) to be associated with contraceptive use among female head‐porters.

**Conclusion:**

Fourteen percent had experienced sexual exploitation post‐migration, the first sexual encounter of one‐third of participants were nonconsensual, 19% had sex at or before 16 years, and 72% were aware of contraception. Reproductive experiences such as gravidity and sexual debut (age at first sex) have a significant influence on the use of contraception.

## 1 INTRODUCTION

The Sustainable Development Goals (SDGs) acknowledge the importance of access to sexual and reproductive health care. SDGs 3.7 and 5.6 place a premium on ensuring that everyone has access to family planning, information, and education, as well as other sexual and reproductive health services.^1^ In line with the agenda 2030, there is an urgent need for global sexual and reproductive health education and promotion among migratory adolescent girls. This is necessary since migration is a significant worldwide policy issue and increases the risk of poor outcomes for females' sexual and reproductive health.^2^, ^3^ Globally, there are 740 million internal migrants, which makes this demand even more pressing. In urban areas of developing countries, adolescent girls make up 80% of this group.^4^, ^5^

Migration has become an essential part of human social life due to economic disparities across the globe, regions, or nations. Even in the same country, inequitable distribution of socioeconomic and educational opportunities causes people to move from one part of the country to the other.^6^ Internal migration to Ghana occurs in all directions.^7^ Migration from the north to the south, however, is more common and is associated with health and socioeconomic implications. Internal migration is so common that almost every northern woman is expected to migrate south at least once in their lifetime in search of better economic future.^8^

Female head‐porters are a cohort of women who have migrated from their rural towns into commercial/urban cities in search of better economic opportunities. This internal migration involves young women moving from their places of origin or livelihood to destinations across geographical boundaries within the same country.^9^

Female head‐porters are locally referred to as *Kayayei* (*Kayayo* for singular). “Kaya” in Hausa means luggage, load, or goods, while “yei” in Ga language means females. Their name literally means a young woman who carries luggage, goods, or a load for a fee. They usually help carry heavy loads of goods from bus stations and markets to various destinations for a negotiated fee.^8^ They therefore aggregate at the large market centers of the city during working hours awaiting offers. Most of these head‐porters are young females with low level of education.^10^ Half of these migrants have either no education at all or have had only primary education^9^ and want to save money for future investment or marriage. Previous studies show most of these migrants do not intend to be in the business for long but see head porterage as a stepping stone to better investment in the future.^11^

Movement of these young women has both positive and negative effects on their personal lives and the society at large. Even though some may have an improvement in their personal incomes, they may suffer from many dire consequences which may affect their future and health.^12^ These negative consequences and outcomes of migration may impact negatively on their reproductive health and rights as well. There is a perceived increase in unstable sexual unions, unintended pregnancies, and unsafe abortions among these women compared to the general population.^13^ It is also believed that access to effective and safe contraception is low among them. Unintended pregnancy and abortion have been closely linked to contraceptive use and certain socio‐demographic indicators such as literacy and level of urbanization.^14^

Female head‐porters may not have access to modern contraceptives as they are usually beyond the reach of reproductive health advocacy programs. Previous studies show that among *Kayayei*, sexual violence was a common phenomenon, and even for consensual sex, their partners often object to the use of contraception.^15^ In 2015, the Government of Ghana drafted a policy whereby there was commitment by all stakeholders to increase the modern contraceptive prevalence rate to 30% among married women and 40% among unmarried sexually active women by 2020, according to the Ghana Family Planning Costed Implementation Plan in 2016–2020. There was no national survey at the end of the program to evaluate the outcome of the costed implementation program. However, according to the recent Ghana Maternal Health Survey which was done in 2017, the modern contraceptive rate was 25% among married women and 31% among unmarried sexually active women. These prevalence rates, though an improvement, still fall below expectations. According to the Metro Health Directorate at the study site, task sharing has been used to provide contraceptive services to difficult‐to‐reach population such as female head‐porters. Community health nurses who visit homes for immunization are therefore trained and mandated to offer contraceptive services as well. Most public health studies among the female head‐porters in the Kumasi Metropolis had been based on malaria and musculoskeletal pains with less emphasis on their reproductive health experiences.^16^ The few studies on reproductive health among female head‐porters were mostly qualitative studies. Data pertaining to the reproductive experiences of these vulnerable women is limited, especially in Kumasi. This study therefore assesses reproductive experiences and factors influencing contraceptive use among Ghanaian female head‐porters.

## 2 MATERIALS AND METHODS

### 2.1 Study design

This was a cross‐sectional analytical study that took place from January to May 2021. This was the most feasible and cost‐effective design to answer study questions and evaluate study objectives within the given time period. The study determined both independent and dependent factors at the same time.

### 2.2 Study site

This study was conducted in the Asokore Mampong Municipality and Kumasi Metropolis. Kumasi is the second largest city in the middle half of Ghana. Market centers in the metropolis used for this study included Bantama market, Kejetia market, and Racecourse market. The residencies of female head‐porters are in the slums and deprived areas of the city. Some of these areas do not have potable water, access roads, health facilities, or security. Their living and sleeping places do not provide privacy for these girls. The majority of the places of residence of female head‐porters have no access to mass media. The market centers where they trade have information centers. These information centers, however, hardly provide information on reproductive health but are usually used for advertisement of goods sold at the market and for religious activities.

### 2.3 Study population

The study's population included female head‐porters whose ages were between 15 and 49. They are also referred to as “Kayayei” or “paa‐o‐paa” in the local dialect. They have low socioeconomic status and thus migrate to the cities in search of greener pastures. In their bid to make some income, they are compelled to carry head‐loads of goods from place to place around markets and bus stations for a fee.

### 2.4 Inclusion/exclusion criteria

This study included female head‐porters within the reproductive age of 15–49 years.

However, female head‐porters who speak languages that cannot be understood by the research team, those who have spent less than a year in the city post‐migration, and those unable to give informed consent to participate in the study were excluded from the study. Also excluded from the study are those not certain of their age.

### 2.5 Sample size calculation

Sample size was obtained using the formula:

$$n=\frac{Z^{2}\;\mathrm{PQ}}{d^{2}},$$

where *n* is the minimum sample size, *P* is the estimated value for the proportion of contraceptive use among Ghanaian females = 18.3%,^17^ *Q* = 1 − *P*, *Z* = z value at 95% confidence (1.96), and *d* is the margin of error (0.05).

$$n\;(\text{Minimum number of participants})=\frac{1.96^{2}(0.183)\left(1-0.183\right)}{0.05^{2}}=229.74$$

Taking into consideration 15% nonresponse rate and to increase statistical power, 280 participants were recruited for the study.

### 2.6 Ethical consideration

Ethical approval for this study was granted by the Committee on Human Research, Publication and Ethics, School of Medical Sciences, Kwame Nkrumah University of Science and Technology (CHRPE/SMS/KNUST) and Komfo Anokye Teaching Hospital (KATH). Consent was also sought from the Kumasi Metropolitan Assembly and the Metro Health Directorate. Moreover, individual consent was sought from each client before enrolling them as study participants.

### 2.7 Sampling and data collection

Convenience sampling was used to select certain markets and residential places for the study. This sampling technique helped us locate market centers with the most intense head porterage activities. Convenience sampling also enabled the research team to identify residencies with high numbers of female head‐porters. Since there was no sampling frame for female head‐porters for random sampling, respondents were recruited consecutively till we achieved the required sample size of 280.

Data were collected with semi‐structured questionnaire (Supporting Information: Appendix S1). The questions were closed and open‐ended with options provided for certain portions. The research team read the questions out to the understanding of the study participants and answers provided were transferred to the questionnaire. Pretesting was done in the Obuasi municipality, an hour and half's drive from the chosen study site. Necessary corrections were made to the questionnaire before the final application.

### 2.8 Data management and statistical analysis

Collected data were entered, cleaned, and coded into Microsoft Excel spreadsheet software. They were then transferred to Stata version 16.0 (StataCorp LLC) for analysis and interpretation. The electronic data were strictly kept under passwords available to the principal investigator and ethics committee chairman alone to ensure confidentiality.

Data were summarized by descriptive statistics; for continuous variables, mean and median were determined. Proportions and percentages were used to estimate the prevalence of discrete predictors and outcome measures. Chi‐square or Fischer's square was used to determine the level of significant associations between the study variables and contraceptive use. A *p*‐value of <0.05 was considered statistically significant.

## 3 RESULTS

### 3.1 Sociodemographic characteristics

Of the 280 study participants included in the statistical analysis, most were between the ages of 15–19 years (37.1%). Majority of participants earned above the minimum daily wage of ≥Gh₵12.00. Most (84.3%) had no extra income activities. Half of the participants had no formal education (48.9%); 53.9% were married; 54.2% resided at Racecourse; and 45.0% had migrated from Northern, North East, or Savannah region. Moreover, the majority belonged to the Islamic religion (65.0%) and had indoor shelters (Table 1).

**Table 1:** Sociodemographic characteristics of study participants.

Variables	Frequency (*n* = 280)	Percentage (%)
**Age group (years)**		
15–19	104	37.1
20–24	54	19.3
25–29	56	20.0
30–34	22	7.9
35–39	26	9.3
>39	18	6.4
**Extra income activities**		
None	236	84.3
Farming	8	2.9
Petty trading	11	3.9
Cleaning	12	4.3
Other^a^	13	4.6
**Highest education level**		
No formal education	137	48.9
Primary level	53	18.9
Junior high level	48	17.2
Senior high level	42	15.0
**Marital status**		
Married	151	53.9
Unmarried	121	43.2
Cohabiting	8	2.9
**Religion**		
Islam	182	65.0
Christianity	68	24.3
Traditional	30	10.7
**Level of income**		
Low (<12ghc daily)	65	23.2
High (≥12ghc daily)	215	76.8
**Region of migration**		
Ashanti region	6	2.1
Bono region	2	0.7
Northern/North East/Savannah regions	126	45.0
Upper West region	96	34.3
Upper East region	48	17.2
Western region	2	0.7
**Residence**		
Dagomba line	71	25.4
Racecourse	152	54.2
Suame	57	20.4
**Type of shelter**		
Indoors	263	93.9
Open (outdoor)	17	6.1

*Note*: Author's construct (field study, 2021).

^a^ Other extra income activities: landscaping and food vendoring.

### 3.2 Sexual and reproductive experiences

From the study, about 14% had experienced sexual exploitation post‐migration. About one‐third of study participants report that their first sexual encounter was nonconsensual. Furthermore, 19% had sex at or before 16 years and 72.1% were aware of contraception (Table 2).

**Table 2:** Sexual and reproductive experience of participants.

Variable	Frequency (*n* = 280)	Percentage (%)
**Gravidity**		
0	78	27.9
1–2	94	33.6
3–4	52	18.5
>4	56	20.0
**Parity**		
0	102	36.4
1–2	85	30.4
3–4	53	18.9
>4	40	14.3
**Sexual exploitation post migration**		
Yes	39	13.9
No	241	86.1
**Ever had sex**		
Yes	202	72.1
No	78	27.9
**Nonconsensual sexual debut** a		
Yes	76	37.6
No	126	62.4
**Age at first sex** a		
< 16years	23	19.0
≥ 16years	98	81.0
**Awareness of contraceptives**		
Yes	202	72.1
No	78	27.9

^a^ Variables with missing data.

### 3.3 Contraceptive among female head‐porters

**Figure 1:**
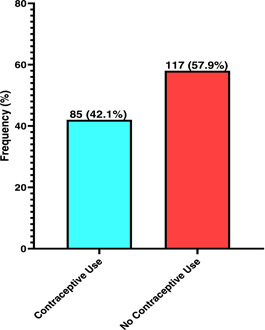
Proportion of contraceptive use among female head‐porters *N* = 85 (42.1%) women with contraceptive use *N* = 117 (57.9%) women with no contraceptive use.

Of the 202 participants aware of contraception, 85 (42.1%) had history of contraceptive use while more than half (57.9%) had no history of contraceptive use (Figure 1). The commonly used contraception in descending order of use were injectables (47%), implants (26.8%), and pills (19%).

**Figure 2:**
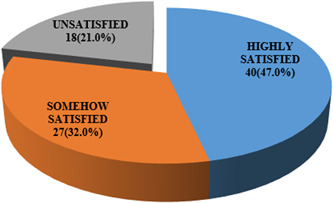
Level of satisfaction with contraceptive use among female head‐porters. The highly satisfied are comfortable with use of method without reservation. The somehow satisfied are happy with current method but will prefer another method or an advanced form of the method in use due to side effects or difficulty associated with its use.

Moreover, of the 85 respondents who had used contraceptives before, 47% were highly satisfied with the use of particular method, while almost one‐third (32.0%) were somehow satisfied and 21% were unsatisfied (Figure 2). The highly satisfied group is comfortable with the method without reservation. Somehow satisfied are happy with the method but would like a better method or an improved version if available due to the side effects of the current method or difficulty associated with its use.

This study found the age group of female head‐porters (*p* = 0.005), marital status (*p* < 0.0001), and religion (*p* = 0.029) to be significantly associated with contraceptive use among female head‐porters (Table 3).

**Table 3:** Sociodemographic factors associated with contraceptive use among female head‐porters.

	Contraceptive use		
Variable	Yes (*n* = 85)	No (*n* = 117)	*p* Value
**Age group (years)**			**0.0050**
15–19	11 (12.20)	35 (28.28)	
20–24	23 (26.83)	29 (24.74)	
25–29	24 (29.27)	11 (12.12)	
30–34	14 (15.85)	19 (15.15)	
35–39	7 (8.54)	13 (11.11)	
>39	6 (7.32)	10 (8.59)	
**Marital status**			**<0.0001**
Unmarried	21 (24.39)	61 (52.53)	
Married	59 (69.51)	54 (45.96)	
Cohabiting	5 (6.10)	2 (1.51)	
**Religion**			**0.0290**
Muslim	65 (76.47)	70 (60.10)	
Christian	13 (15.29)	33 (28.28)	
Traditional	7 (8.24)	14 (11.62)	
**Level of education**			0.6000
No formal education	41 (48.78)	56 (47.47)	
Primary level	20 (23.17)	20 (17.68)	
JHS level	14 (15.85)	22 (18.69)	
SHS level	10 (12.20)	19 (16.16)	
**Income level**			0.2010
<Minimum daily wage	26 (30.49)	46 (38.89)	
≥Minimum daily wage	59 (69.51)	71 (61.11)	
**Type of shelter**			0.6930
Indoor	81 (95.12)	110 (93.94)	
Open	4 (4.88)	7 (6.06)	
**Area having hospital**			0.4790
No	38 (44.70)	47 (40.20)	
Yes	47 (55.30)	70 (59.80)	
**Local hospital education programs**			0.3190
No	39 (45.80)	62 (52.90)	
Yes	46 (54.10)	55 (47.10)	
**Media access at home**			0.3100
No	50 (58.80)	77 (65.80)	
Yes	35 (41.20)	40 (34.20)	

*Note*: Bold = significant variables.

However, the educational level (*p* = 0.600), income level (*p* = 0.201), type of shelter (*p* = 0.693), hospital available at area (*p* = 0.479), local hospital education programs (*p* = 0.319), and media access at home (*p* = 0.310) were not significantly associated with contraceptive use among female head‐porters.

### 3.4 Influence of sexual and reproductive factors on contraceptive use

Table 4 shows the influence of sex and obstetric factors on contraceptive use among female head‐porters. This study found gravidity of participants (*p* < 0.0001), parity of participants (*p* < 0.0001), number of sexual partners post‐migration (*p* = 0.008), and age of first sex (*p* = 0.033) to be significantly associated with contraceptive use among female head‐porters.

**Table 4:** Sex and obstetric factors associated with contraceptive use among female head‐porters.

	Contraceptive use		
Variable	Yes (*n* = 85)	No (*n* = 117)	*p* Value
**Gravidity**			**<0.0001**
0	29 (34.15)	30 (25.25)	
1–2	9 (10.98)	50 (42.93)	
3–4	22 (25.60)	18 (15.66)	
>4	25 (29.27)	19 (16.16)	
**Parity**			**<0.0001**
0	38 (45.12)	38 (32.83)	
1–2	9 (10.98)	45 (38.38)	
3–4	18 (20.73)	21 (18.18)	
>4	20 (23.17)	13 (10.61)	
**Sexual exploitation**			0.5690
No	75 (87.80)	100 (85.35)	
Yes	10 (12.20)	17 (14.65)	
**Nonconsensual sexual debut**			
Yes	36 (42.35)	40 (34.19)	0.2370
No	49 (57.65)	77 (65.81)	
**Number of sexual partners post‐migration**			**0.0080**
0	27 (31.71)	63 (53.54)	
1	56 (65.85)	52 (44.44)	
2	2 (2.44)	2 (1.72)	
**Age at first sex** a			**0.0330**
<16 years	20 (39.22)	3 (4.29)	
≥16 years	31 (60.78)	64 (95.71)	

*Note*: Bold = significant variables.

^a^ Variables with missing data.

On the contrary, sexual exploitation (*p* = 0.569) and nonconsensual sexual debut (*p* = 0.237) were not significantly associated with contraceptive use among female head‐porters.

## 4 DISCUSSION

Of the 280 participants included in this study, 202 (72%) were aware of contraception which is lower than the 99% awareness in the country according to the 2014 Ghana Demographic and Health Survey. The study found the age group of female head‐porters (*p* = 0.005), marital status (*p* < 0.0001), and religion (*p* = 0.029) to be significantly associated with contraceptive use among female head‐porters. Furthermore, gravidity (*p* < 0.0001), parity (*p* < 0.0001), number of sexual partners post‐migration (*p* = 0.008), and age of first sex (*p* = 0.033) were significantly associated with contraceptive use among female head‐porters.

The finding of 42.1% of contraceptive use among Ghanaian female head‐porters is much higher compared to studies by Beson et al.,^18^ and Bawah et al.,^19^ who reported contraceptive use between 21% and 13%, respectively. The observed higher difference between the present and previous studies may be due to differences in the study area as geographical location may have an impact on access to contraceptive use. The Beson study was done in the Southern coast of Ghana, while the Bawah study was in the Upper East Region of the country.

We observed age group, marital status, and religion influencing contraceptive use among female head‐porters. These findings are in agreement with studies of Beson et al.,^20^ Anguzu et al.,^21^ and Ekorinyang,^22^ which showed that age, marital status and religion influence the use of contraceptives. However, this study could not establish the strength and direction of the association between these variables. The fears of becoming pregnant or having sexually transmitted infections, as well as being chastised by one's religious affiliation and doctrines, may encourage contraceptive use. Contraceptive use may therefore be prioritized over religious views, as long as they are aware of the benefits, such as preventing unwanted pregnancies and sexually transmitted diseases.

Furthermore, this study found gravidity, parity, number of sexual partners post‐migration, and age of the first sex influencing contraceptive use among female head‐porters. This is in line with studies done by Park and Kim,^23^ de Vargas Nunes Coll et al.,^24^ Manlove et al.,^25^ and Magnusson et al.^26^ Aside from nulliparous women, contraception use increased with increasing parity. This could be attributed to individuals being able to make decisions on parenthood after their first experience of parity. Female head‐porters are therefore more likely to resort to contraception after having achieved pregnancy a couple of times. Those who have never been pregnant are less likely to use contraception, thus making them vulnerable to sexually transmitted infections and unintended pregnancies. The study's findings showed the age of the first sex and the number of sexual partners post‐migration as having significant associations with contraceptive use. This finding is similar to that of Manlove et al.^25^ and Magnusson et al.^26^

The findings of this study show reproductive educational campaigns should be increased to enhance contraceptive use among female head‐porters. This will help reduce the prevalence of unintended pregnancies and sexually transmitted infections. Moreover, alternative job opportunities should be provided to prevent female head‐porters resorting to dependencies which may lead to sexual exploitation.

## 5 LIMITATIONS

The study has no controlled group. Moreover, data were collected by both convenience sampling and consecutive recruitment due to the absence of a formal sampling frame for study participants. There are also missing data for certain variables largely because certain information could not be ascertained from study participants.

## 6 CONCLUSION

Contraceptive use among female head‐porters was comparatively high. The majority of women (female head‐porters) who had ever used contraception were satisfied with its use. Fourteen percent had experienced sexual exploitation post‐migration. The first sexual encounter of about one‐third was nonconsensual. Nineteen percent had sex at or before 16 years. Awareness of contraception was 72%.

Factors such as gravidity, parity, the number of sexual partners post‐migration, and age of first sex significantly influenced the use of contraceptives among female head‐porters.

## AUTHOR CONTRIBUTIONS

**Seth Amponsah‐Tabi**: Conceptualization; investigation; methodology; project administration; resources; supervision; writing—review and editing. **Kwadwo Sarbeng**: Supervision. **Edward Dassah**: Supervision; validation. **Amponsah Peprah**: Conceptualization; project administration; validation; writing—original draft. **Gerald Owusu Asubonteng**: Supervision; writing—review and editing. **Frank Ankobea**: Supervision; validation. **Stephen Opoku**: Data curation; formal analysis; investigation; writing—review and editing. **Ebenezer Senu**: Formal analysis; methodology; writing—review and editing. **Emmanuel S. K. Morhe**: Supervision; validation; writing—review and editing. **Kwabena Antwi Danso**: Supervision; visualization; writing—original draft.

## CONFLICT OF INTEREST STATEMENT

The authors declare no conflict of interest.

## ETHICS STATEMENT

The study was approved by the Committee on Human Research, Publication and Ethics, School of Medical Sciences, Kwame Nkrumah University of Science and Technology (CHRPE/SMS/KNUST) and Komfo Anokye Teaching Hospital (KATH). Consent was also sought from the Kumasi Metropolitan Assembly and the Metro Health Directorate. Moreover, individual consent was sought from each client before enrolling them as study participants.

## TRANSPARENCY STATEMENT

The lead author Seth Amponsah‐Tabi affirms that this manuscript is an honest, accurate, and transparent account of the study being reported; that no important aspects of the study have been omitted; and that any discrepancies from the study as planned (and, if relevant, registered) have been explained.

## Supporting information

Supporting information.

## Data Availability

All data generated or analyzed during this study are included in this article and its Supporting Information files and can be requested from the corresponding author.
