# Supplementary material for: Reproductive experiences and factors influencing contraceptive use among female head‐porters in Ghana: A cross‐sectional study
Source: Health Sci Rep. 2023 Jun 1;6(6):e1298. doi: 10.1002/hsr2.1298 (PMC10233331; doi:10.1002/hsr2.1298)
Supplement: Supplementary file 1 — Supporting information. [file HSR2-6-e1298-s001.docx]

SUPPLEMENTARY FILES

QUESTIONNAIRE

APPENDIX I

QUESTIONNAIRE

| 1. **Study ID** .............................................................. |
| --- |

| 1. What is your age (*in years*) ..................................... [__][__] |
| --- |

| **Parity Now**.............................................. [__][__]  Gravidity……………………………. |
| --- |

| **What is your occupation** (please circle as appropriate)  Head potter? YES [ ] NO [ ]  Other? ……  Unemployed……………………………………………………………….1  Farmer……………………………………………………………………..2  petty trading……………………………………………………………....3  civil/public servant…………………………………………………..........4  artisan……………………………………………………………………..5  other………………………………………………………………………6 |
| --- |
|  |

| **Highest education level** (please circle appropriate number):  None………………………0  Primary…………………….1  JSS………………………….2  Secondary………………… 3  Tertiary…………………… 4 |
| --- |

| **What is your marital status** (please circle appropriate number)?  Single…………………….1  Married……………………2  Divorced………………….3  Widowed…………………4  Co-habiting……………….5 |
| --- |
| Religion  Christianity………………………..  Islam……………………………….  Traditional belief………………….. |

Did you migrate or you were born here? Yes [ ] No [ ]

If yes to Q1, where did you migrate from before settling in Kumasi………….?

For how long have you been in Kumasi? ………………

Why did you migrate down south? Economic [ ] education [ ] Apprenticeship [ ]other …….

How long have you been doing this Kayayei ……….?

Where do you live/sleep after work (residence)………………?

Type of shelter or accommodation: Open……………Indoor…………

How much do you pay for the accommodation…………………….?

SEXUAL AND REPRODUCTIVE ISSUES

1a. Age at first sexual intercourse………………………………………

1b. describe the circumstances to your first sex intercourse (consensual or by coercion should be in the mind of the interviewer)……………………..

1c. Have you ever experienced any kind of sexual exploitation? Yes [ ] No [ ]

1d. if yes to question 1c, which of the exploitations Rape [ ] harassment [ ]

sex for favour [ ]

Who was responsible? Partner/ family member/friend/ caretaker/other (specify)……

How many sexual partners have you had since you migrated to down south? …

How many sexual partners do you have now?.......................................

CONTRACEPTION

1. Are you aware of any contraception method?.............................................................

2.Which one of the methods are you aware of?.......................................... (study participant can choose more than one answer for question 16)

Combined oral contraceptive pill

Progesterone only pills

Condoms

Jadelle/implanon NXT

Depo provera injections

Norigynon injections

Intrauterine device or systems

Tubal ligation

Vasectomy

3.Were you counseled of how to use a particular method of choice? Yes [ ] No [ ]

4.Were you educated on possible side effects? Yes [ ] No[ ]

5.What was your source of information concerning family planning? (can choose more than one answer) Media [ ] health workers [ ] church/Mosque [ ] Family and friends [ ]

6. Have you ever used any form of contraception? Yes [ ] No [ ]

If no, jump to Q10

7. Which method? (as many as can recall) .............................................................................................................

8.What was your level of satisfaction with its use? Highly Satisfied ] somehow satisfied [ ] Unsatisfied [ ]

9.If unsatisfied, why? Method failure [ ] side effects [ ] difficult to use [ ] others [ ]………

10.If no to question 6: Do you have intentions to use any modern contraception? Yes [ ] No[ ]

11. When do you intend to use it?.......

12.Why have you not used a method despite your intentions?............................

13. Have you ever had an education about reproductive health? Yes [ ]No [ ]

14. What do you remember about the educational talk? ………………………..

15. What/ who was the source of your education……………………………………….

16. Where do you usually obtain or purchase your contraceptive method from?................

17.How will you rate your accessibility to your choice of contraception? Always accessible/ oftenly accessible/ accessible/ difficult to access/inaccessible

PERSONAL, SOCIAL AND COMMUNITY CHARACTERISTICS

1. How much money do you make daily from this business?..........

2. Do you undertake other income generating activities to sustain yourself?........

3. How much do you spend daily for your upkeep or your family’s upkeep…….

4. Who do you turn to when hard pressed for money?...............................

5. Are you covered by National health insurance?.............................

6. Do you pay additional money when you visit the hospital even as NHIS card holder? Yes[ ]No [ ]

7. What are those additional monies meant to pay for?...........................

8. What do leaders in your community do or how do they react when a teenager becomes pregnant unexpectedly? Nothing [ ] Summons and cautions the teenager [ ] punishes teenager [ ]

9. Do health workers or other groups (NGOs) visit your community to educate you and your neighbors on reproductive health (sex, contraception, STIs, pregnancy and abortions) Yes [ ]No [ ]

10. If yes, how many times in a year?............................

11. Do you have a hospital, clinic and health post in your area where you live Yes [ ] No [ ]

12. If yes to question 11, does this health institution educate the people on reproductive health issues? Yes [ ] No [ ]

13. Do you have access to mass media (radio or TV) at your place of residence? Yes [ ] No [ ]

14. Do you hear of any educational programs from the media about reproductive health (sex education, contraception, STIs, pregnancy and abortions) Yes [ ] No [ ]

15. If yes to question 14, how many times in month do you hear of reproductive health education from the media………….

16. Have you had reproductive health education from NGOs, civic rights groups or NCCE? Yes[ ] No[ ]
